# Diagnosing drug-induced AIN in the hospitalized patient: A challenge for the clinician 

**DOI:** 10.5414/CN108301

**Published:** 2014-04-02

**Authors:** Mark A. Perazella

**Affiliations:** Section of Nephrology, Yale University School of Medicine, New Haven, CT, USA

**Keywords:** urine microscopy, eosinophiluria, leukocytes, white blood cell cast, acute kidney injury, acute interstitial nephritis, acute tubular necrosis

## Abstract

Drug-induced acute interstitial nephritis (AIN) is a relatively common cause of hospital-acquired acute kidney injury (AKI). While prerenal AKI and acute tubular necrosis (ATN) are the most common forms of AKI in the hospital, AIN is likely the next most common. Clinicians must differentiate the various causes of hospital-induced AKI; however, it is often difficult to distinguish AIN from ATN in such patients. While standardized criteria are now used to classify AKI into stages of severity, they do not permit differentiation of the various types of AKI. This is not a minor point, as these different AKI types often require different therapeutic interventions. Clinicians assess and differentiate AIN from these other AKI causes by utilizing clinical assessment, various imaging tests, and certain laboratory data. Gallium scintigraphy has been employed with mixed results. While a few serum tests, such as eosinophilia may be helpful, examination of the urine with tests such as dipstick urinalysis, urine chemistries, urine eosinophils, and urine microscopy are primarily utilized. Unfortunately, these tools are not always sufficient to definitively clinch the diagnosis, making it a challenging task for the clinician. As a result, kidney biopsy is often required to accurately diagnose AIN and guide management.

## Introduction 

**Figure 1:**
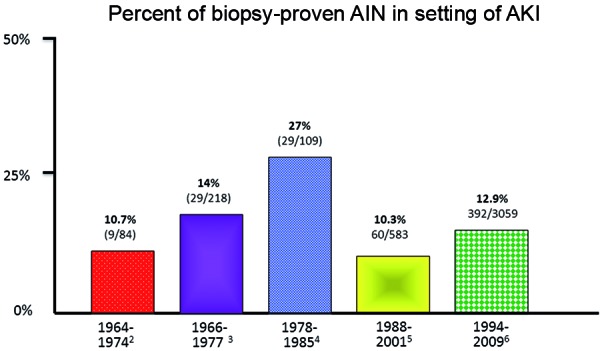
Prevalence of AIN in patients with acute kidney injury. AIN = acute interstitial nephritis.

Clinicians commonly encounter acute kidney injury (AKI) in patients admitted to the general hospital wards and the intensive care units [1]. The majority of hospital-acquired AKI cases are due to either prerenal AKI or acute tubular necrosis (ATN); however, unrecognized acute interstitial nephritis (AIN) is likely the third most common cause [2]. In fact, AKI without an obvious cause is attributed to biopsy-proven AIN in anywhere from 10 to 27% (Figure 1) of patients [2, 3, 4, 5, 6]. As such, healthcare providers must be knowledgeable in the diagnostic evaluation of AKI to be able to differentiate these various entities. This is particularly important as AKI is a growing problem in the hospital and its incidence continues to increase [1]. Similarly, the prevalence of AIN, primarily due to drugs (> 85%), also appears to be increasing as a cause of hospital-acquired AKI [6]. 

Since AKI is linked to untoward outcomes such as incident and progressive chronic kidney disease (CKD), end-stage renal disease (ESRD), and death, it is all the more important to rapidly diagnose and treat the underlying condition [7]. To this point, the inability to temper the adverse outcomes associated with AKI may be related to a combination of late recognition and delayed initiation of directed therapeutic strategies. In the case of AIN, culprit drug withdrawal and corticosteroid therapy may salvage renal tissue by reducing the amount of tubulointerstitial fibrosis that develops [8, 9]. 

In current times, evaluation of AKI patients has become more standardized through the use of definitions such as the Risk-Injury-Failure-Loss-End Stage (RIFLE), Acute Kidney Injury Network (AKIN), and Kidney Disease Improving Global Outcomes (KDIGO) AKI criteria to diagnose and classify this entity [1, 7, 10]. These criteria, however, do not permit differentiation of the various types of AKI, including prerenal AKI, ATN, and AIN, which ultimately require different management approaches. 

In differentiating AIN from these other causes of AKI, clinicians utilize a variety of clinical tools such as history, physical examination, imaging tests, and certain laboratory data. While clinical history and exam are extremely important, additional diagnostic tests are often required to more accurately distinguish these entities. Gallium scintigraphy, and more recently positron emission tomography (PET) scan have been employed. A few serum tests may be helpful, but for the most part, urinary tests are utilized to differentiate AIN from these common causes of hospital-acquired AKI. These consist primarily of dipstick urinalysis, urine chemistries, urine eosinophils, and physician-performed urine microscopy. After briefly touching on history, examination, and serum tests, this *Clinical Nephrology Perspectives* article will focus on the utility (and futility) of the major tests available and employed to diagnose AIN. Ultimately, kidney biopsy is required to accurately make a diagnosis and guide therapy. 

## Clinical history and physical exam 

Most critical in the clinical evaluation of the patient where AIN is considered part of the differential diagnosis is determining exposure to a suspect medication. While any drug may cause AIN, classic and common agents are certain antimicrobial agents (β-lactams, sulfonamides, quinolones, anti-viral agents), anti-ulcer agents (proton pump inhibitors [PPIs], H_2_-antagonists), non-steroidal anti-inflammatory drugs (NSAIDs), anticonvulsants, and allopurinol [8, 9]. Table 1 provides a list of selected medications that are associated with AIN. 

Non-specific symptoms are generally noted with AIN [8, 9]. These include generalized malaise, fatigue, weakness, anorexia, and nausea. At times patients will describe myalgias and arthralgias, flank pain, and “feeling feverish”. A pruritic skin rash may be another complaint, raising suspicion for an allergic or drug-related process. However, none of these are particularly specific to AIN and may be seen in many hospitalized patients with or without AKI. 

A physical examination finding that sometimes points towards AIN is a low grade or spiking fever that occurs in the absence of documented infection. However, it is often difficult to sort this out in hospitalized patients that are receiving antibiotics for infection and those with invasive devices in place, such as peripheral or central vein catheters, and indwelling bladder catheters. In addition, fever is not uniformly present, although it commonly occurs with AIN from methicillin and other penicillin derivatives [4, 5, 6, 7, 8, 9, 10, 11, 12, 13, 14, 15, 16]. 

A classic drug eruption, typically morbilliform and involving the trunk, can be very helpful in suggesting drug-related AIN. However, it is not a sensitive finding and is frequently not present even in the setting of rip-roaring AIN. In general, drug rash is reported in 15 – 50% of AIN cases, is more likely with drugs that cause a hypersensitivity reaction (β-lactams, sulfonamides, phenytoin), and is rarely seen (or completely absent) with drugs such as PPIs and NSAIDs [4, 11, 17]. Palpably enlarged, tender kidneys have been described but are rarely found on exam [8, 9]. Thus, in the absence of culprit drug exposure and a classic drug eruption, it is difficult to place AIN at the top of the differential for hospital-acquired AKI in the absence of other supportive data. 

## Serum tests 

### Serum eosinophils 

The blood test most helpful in raising the specter of drug-induced AIN is an elevated serum eosinophil count. Significant eosinophilia often reflects an allergic drug reaction, and may be very helpful diagnostically for the patient with hospital-acquired AKI [8, 9]. While eosinophilia occurs in other AKI settings such as cholesterol emboli syndrome, vasculitis, and malignancy, these processes are often clinically recognizable [8, 9]. Unfortunately, as with other tests employed in the evaluation of AIN, serum eosinophils are not a sensitive finding. Serum eosinophils may be only modestly elevated or markedly abnormal, at times making up 50 – 75% of the total white blood cell count [18]. As with fever and drug rash, significant eosinophilia in AIN has a wide range, is more common with certain drugs (similar to drug rash), and may be absent even when an eosinophil-dominant AIN is seen on kidney biopsy [4, 14, 17]. Most disappointing is the lack of diagnostic utility of the combination of fever, rash, and eosinophilia for AIN, where the triad is seen in only 5 – 10% of patients with AIN [5, 14]. 

### Other tests 

Anemia is often present in the setting of AIN. However, this blood abnormality is quite nonspecific and widely prevalent in many hospitalized patient, especially those with AKI alone or superimposed on CKD [5]. Anemia likely results from a number of processes including loss of erythropoietin (EPO) production from kidney injury, as well as EPO hyporesponsiveness or resistance from inflammation and/or infection [5]. Liver function tests (LFTs) may also be abnormal with AIN, primarily due to an associated drug-induced hepatitis. However, this finding is exceedingly rare in AIN, and multiple other processes can elevate LFTs in the hospitalized patient. Erythrocyte sedimentation rate and C-reactive protein may also be elevated with AIN, although they are very non-specific findings [5, 17]. These tests are not otherwise useful. Finally, the alert nephrologist may notice a hyperkalemic, hyperchloremic metabolic acidosis, out of proportion to the degree of kidney failure, raising suspicion for associated tubulointerstitial injury [17]. Other patterns of tubulointerstitial injury can be seen such as a Fanconi syndrome, salt-wasting nephropathy, distal renal tubular acidosis, and urinary concentrating defects [17]. 

## Imaging modalities 

### Ultrasound and CT scan 

Kidney imaging with either ultrasonography or computed tomography (CT) scan provides structural information such as kidney size and number, cortical echogenicity, and presence or absence of hydronephrosis, cysts, masses, or stones. Thus, the utility of these modalities lies with their exclusion of other causes of AKI. While enlarged, swollen kidneys with increased echogenicity on ultrasound are often seen with AIN, this finding is not specific for AIN and can be seen with acute glomerulonephritis, infiltrative diseases, ATN and other etiologies of AKI [8, 9]. In one report, renal volume increased by 200% with AIN [19], presumably related to cellular infiltration and edema. Similarly, CT scan may show renomegaly in the setting of AIN, but this test has the same limitations as renal ultrasound. Overall, these findings are neither sensitive nor specific for AIN; these studies are useful mainly to exclude urinary tract obstruction. 

### Gallium scintigraphy 

Imaging of the kidneys with ^67^gallium scan has been employed in the evaluation of AIN for the past 30 years [20, 21]. Kidneys with AIN enhance as a result up the binding of ^67^gallium to lactoferrin, which is produced and released by leukocytes within the interstitium [20]. In addition, lactoferrin is found on the surface of invading inflammatory cells, primarily lymphocytes, and also binds gallium [20]. Thus, gallium would be expected to enhance kidneys with AIN. An investigation in rats demonstrated that ^67^gallium scanning was highly accurate in differentiating experimentally induced AIN from both drug-induced ATN and normal rat kidneys [20]. In humans, there have been both promising study results as well as suboptimal test performance with this modality. An early study revealed excellent sensitivity (11/11, 100%) in patients with biopsy-proven AIN [21]. However, subsequent studies have demonstrated lower sensitivities of 58% [22] and 69% [23] with a test specificity of only 50 – 60% [14, 17]. Positive scan results have been seen with other inflammatory conditions such as pyelonephritis, renal atheroemboli, and glomerulonephritis, as well as with ATN and normal kidney tissue on biopsy [14, 17]. Tracer uptake in the kidneys is measured at 48 – 72 hours following ^67^gallium injection, is most often graded on a scale of 0 to 3+, and is compared to the intensity in the spine [21]. In general, a scan that is considered positive and indicative of AIN requires at least 2+ intensity in the kidneys. One situation where renal scanning with ^67^gallium scintigraphy may be useful is in differentiating AIN from ATN when kidney biopsy is contraindicated or refused by the patient. However, the limitations of this test must be known prior to employing it in such patients. 

### FDG-PET scan 

**Figure 2:**
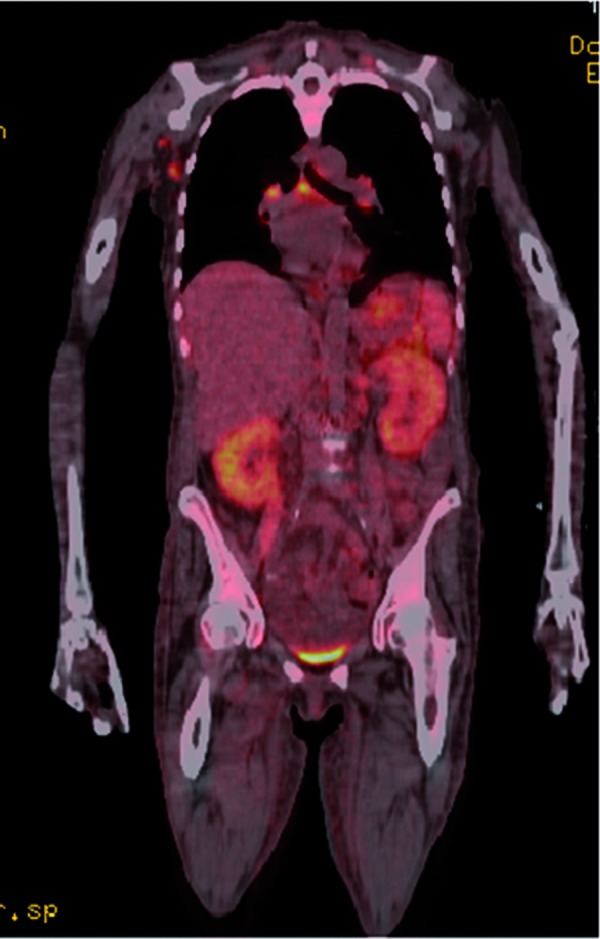
Positron emission tomography (FDG-PET) scan in a patient with acute interstitial nephritis (AIN) in the setting of drug rash with eosinophilia and systemic symptoms (DRESS) syndrome.

Another non-invasive imaging test, employed primarily to evaluate malignant disease, has been recently used to diagnose AIN [24]. A single publication noted a positive 2-[18F] fluoro-2-deoxy-D glucose-positron emission tomography (FDG-PET) scan in 2 patients with severe AKI due to biopsy-proven AIN; one of the patients had a negative gallium scan [24]. In addition, the FDG-PET scan was negative in a patient with AKI from crescentic glomerulonephritis. Repeat FDG-PET scans were negative in the 2 patients with AIN after clinical resolution of kidney injury. My personal experience with this test has been positive with 3 patients having positive FDG-PET scans (Figure 2) in the setting of biopsy proven drug-induced AKI (personal communication). Uptake of tracer in the setting of AIN is based on the premise that FDG accumulates not only in tumor cells but also in the lymphocytes, macrophages, neutrophils and fibroblasts of inflammatory lesions [24]. Thus, this modality should undergo further study to judge its true utility (sensitivity and specificity) for diagnosis of AIN. 

## Urine tests 

### Urinalysis 

Urinalysis is a commonly used diagnostic test in hospitalized patients with AKI. It can provide helpful clues that suggest drug-induced AIN as a diagnostic possibility [14]. Trace, 1 or 2+ proteinuria may be seen on the dipstick, unless there is concomitant glomerular injury (minimal change disease) as can be seen with NSAIDs [14]. Protein : creatinine ratio in spot urine samples generally show levels < 1 g of protein/day, consistent with “tubular” proteinuria [4]. Microscopic, and less commonly macroscopic hematuria is typically seen in < 50% of cases, but is more common, up to 90% with certain drugs, particularly methicillin and the β-lactam class [14, 17]. Urinary leukocytes are considered a common urinary abnormality in the setting of AIN. In early reports on methicillin-associated AIN, leukocytes were noted to be nearly universally present [14, 17]. However, in other forms of drug-induced AIN, leukocytes are noted in 50% or less of cases [12, 15]. A Mayo clinic study noted that ~ 80% of patients with drug-induced AIN had dipstick pyuria [25]. Urinary findings described in 21 cases of biopsy proven drug-induced AIN noted RBCs in 43% and WBCs in 57% of patients, respectively [26]. These studies confirm that hematuria and leukocyturia are common; however, clinicians should not mistakenly exclude AIN as a cause of AKI in the absence of either hematuria or pyuria. 

### Urine chemistries 

Urine concentrations of sodium (Na), urea, and creatinine either examined alone or as fractional excretions (FE) of Na (FENa) or urea (FEurea) are widely used to assess patients with AKI [27]. With some notable exceptions, urine chemistries greatest utility are in distinguishing prerenal AKI from ATN, but are unhelpful for AIN. Patients with AIN have been shown to have FENa values that are both above and below 1% [8, 9]. FEurea has not been widely examined in AIN, but there is no reason to believe it offers any advantage. Thus, urine chemistries are not useful in the evaluation of AIN. 

### Urine eosinophils 

Most clinicians practice with the belief that eosinophiluria is part and parcel of drug-induced AIN. An early description of urinary eosinophils as a potential marker for AIN was noted in 9 cases of methicillin-associated AIN [28], whereas none of 43 patients with AKI from another diagnosis had eosinophiluria. Subsequently, eosinophiluria was described in 6/9 patients with drug-induced AIN [29]. These two small studies promoted more widespread use of eosinophiluria for evaluation of AIN. 

Subsequent work on this subject has noted variable sensitivities and specificities, making the utility of eosinophiluria unclear. An attempt to increase test sensitivity by using Hansel stain rather than Wright stain was pursued [30]. This stain was chosen based on its enhanced accuracy in identifying eosinophils in nasal, bronchial, and ocular secretions of patients with allergic diseases. A small study using this stain noted an improvement in sensitivity to 91%. Further studies of the Hansel stain [31, 32] demonstrated various ranges of sensitivity and specificity (Table 2). Importantly, many processes other than AIN are associated with significant eosinophiluria. These include cystitis or prostatitis, pyelonephritis, atheroembolic disease, ATN, rapidly progressive glomerulonephritis, allergic granulomatosis, bladder tumors, ileal conduits, and asthma, many of which also present with AKI [32]. 

Despite the unclear utility of eosinophiluria, it is frequently ordered in the setting of AKI to evaluate for AIN. My personal experience is that many clinicians order urinary eosinophils in the workup of hospital-acquired AKI, making erroneous decisions based on potentially incorrect results. This stems from inconclusive results generated by small studies with many flaws, in particular the lack of a gold standard for AIN diagnosis. A recent study has shed more light on this test utilizing the largest number of patients with a kidney biopsy gold standard [25]. Over an 18-year period, 566 patients with both urinary eosinophil testing and kidney biopsies performed within the same week were identified. Of these, 91 patients had AIN. Approximately 2/3 of the biopsy-confirmed AIN cases were negative for urinary eosinophils. When urinary eosinophils ≥ 1% was used as a positive test, this assay identified only ~ 31% of AIN cases with a similar positive rate in ATN (29.0%). The sensitivity and specificity for urinary eosinophils (> 1%) were 35.6% and 68.2%, respectively. A 5% urinary eosinophil cut-off improved specificity (91.2%) but with a concomitant decreased sensitivity (23.3%). Thus, urinary eosinophils should no longer be considered a useful marker for AIN. This study provides nephrologists with data to definitively recommend against eosinophiluria as a diagnostic test for AIN [25, 33]. 

### Urine microscopy 

**Figure 3:**
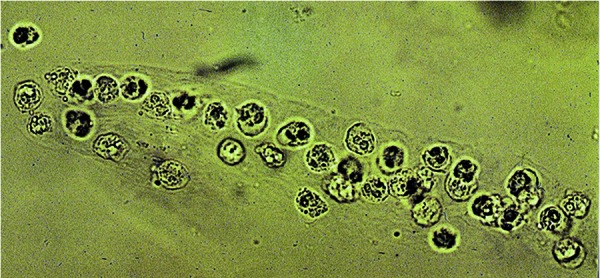
White blood cell cast in the urine of a patient with acute interstitial nephritis (AIN).

A thorough evaluation of the spun urine sediment performed by an experienced nephrologist is considered to be fairly accurate and tantamount to the “liquid biopsy” of the kidney. In addition to free leukocytes and red blood cells (RBCs), white blood cell (WBC) casts (Figure 3) seen in the urine of patients with AKI are highly suggestive of AIN in the absence of pyelonephritis [14, 17]. However, these cellular casts are not necessarily specific for AIN as they may be rarely seen with acute glomerulonephritis and acute papillary necrosis [9]. Other urinary sediment findings also seen with AIN include renal tubular epithelial (RTE) cells, RTE cell casts, and granular casts. Their presence reflects associated tubular cell injury from invading inflammatory cells. Interestingly, a study conducted by an expert in urine microscopy described numerous hyaline and granular casts in 86% (18/21) of patients with biopsy proven drug-induced AIN further supporting renal tubular injury by the underlying inflammatory process [26]. Surprisingly, RBC casts were noted in 26% of cases and WBC casts in only 14% of cases [26]. Thus, clinicians should not mistakenly exclude AIN as a cause of AKI in the absence of pyuria or WBC casts. 

## Kidney biopsy 

**Figure 4:**
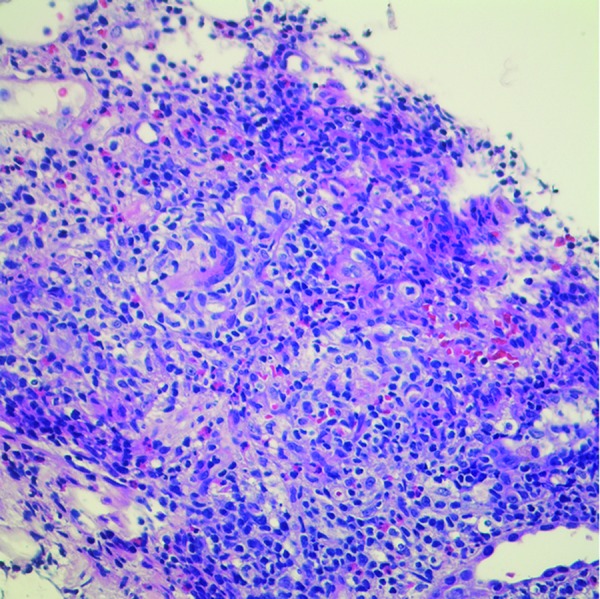
Kidney biopsy of a patient with acute interstitial nephritis (AIN) highlighting the inflammatory interstitial infiltrate with prominent eosinophils.

In the end, a definitive diagnosis of AIN requires kidney tissue. Interstitial inflammation and tubulitis characterize the lesion (Figure 4). The interstitial infiltrate typically contains a predominance of lymphocytes and monocytes, often accompanied by smaller numbers of eosinophils, plasma cells, neutrophils, and histiocytes. The mononuclear component of the infiltrates is composed of primarily T-cells, followed by monocytes, and then B-cells [34]. The composition of the interstitial infiltrate may be helpful in determining the etiology of AIN. For example, a significant component of eosinophils favors drug-induced AIN (Figure 4), whereas neutrophils suggest bacterial infection. However, all cell types may be encountered in drug-induced AIN and in many cases, eosinophils are not identified, especially NSAID-associated AIN. 

Along with interstitial inflammation, AIN is characterized by tubulitis, which represents tubular involvement by interstitial inflammatory cells, primarily lymphocytes. Tubular degenerative changes such as irregular luminal contours, luminal ectasia, prominent nucleoli, cytoplasmic simplification, loss of brush border, and apoptotic figures are also seen in AIN. The cellular infiltrate is associated with interstitial edema early in the process, but over time may transform into interstitial fibrosis and tubular atrophy [34]. Blood vessels and glomeruli are not involved by AIN and are normal unless another process is present. 

## Conclusion 

Drug-induced AIN is a relatively common cause of hospital-acquired AKI. Differentiating AIN from other causes of AKI is often challenging for clinicians. Tools currently utilized for diagnosis include clinical assessment, imaging modalities such as gallium scintigraphy and FDG-PET scan, and a few serum tests, such as eosinophilia. However, urine examination is the test primarily used. Dipstick urinalysis, urine eosinophils, and urine microscopy constitute the most frequently used tests. Unfortunately, these do not always allow a definitive diagnosis, making it a challenging task for the clinician. Most patients require a kidney biopsy to accurately diagnose and manage AIN. 

**Table 1.** Selected drugs associated with acute interstitial nephritis (AIN).

Antibiotics	β-lactam drugs*
	Fluoroquinolones*
	Rifampin*
	Sulfa-based drugs*
	Vancomycin
	Minocycline
	Ethambutol
	Erythromycin
	Chloramphenicol
Antiviral medications	Acyclovir
	Abacavir
	Indinavir
	Atazanavir
GI medications	Proton pump inhibitors*
	Histamine-2 receptor blockers
Analgesics	Nonsteroidal anti-inflammatory drugs*
	Selective COX-2 inhibitors
Anti-seizure drugs	Phenobarbital
	Phenytoin*
	Carbamazepine
Other drugs	Allopurinol*
	5-Aminosalicylates*
	Captopril
	Interferon
	Cyclosporine
	Anti-angiogenesis drugs (tyrosine kinase inhibitors)
	Diuretics

*Most common offending agents.

**Table 2.** Evaluation of eosinophiluria in diagnosis of acute interstitial nephritis (AIN).

Reference	Patients	Sensitivity	Specificity	Other diagnoses
Nolan et al. [30]	N = 92 Hansel stain	10/11 (91%)	69/81 (85%)	ATN (0/30) Pyn (0/10) GN (1/6) RPGN (4/10) Prostatitis (6/10)
Corwin et al. [31]	N = 183 Hansel stain	5/8 (63%)	160/175 (93%)	ATN (1/29) UTI (4/37) DN (4/17)
Ruffing et al. [32]	N = 51 Hansel stain	6/15 (40%)	26/36 (72%)	GN (4/6) CKD (2/5) Pyn (1/2)
**Total**	**326**	**21/34 (62%)**	**255/292 (87%)**	

AIN = acute interstitial nephritis; Eos = eosinophils; UTI = urinary tract infection; CIN = contrast-induced nephrotoxicity; CKD = chronic kidney disease; Pyn = pyelonephritis; GN = glomerulonephritis; RPGN = rapidly progressive glomerulonephritis; ATN = acute tubular necrosis; DN = diabetic nephropathy.
